# Semantics Processing of Conditional Connectives: German *wenn* ‘if’ Versus *nur wenn* ‘only if’

**DOI:** 10.1007/s10936-021-09812-0

**Published:** 2021-10-26

**Authors:** Mingya Liu, Mathias Barthel

**Affiliations:** Department of English and American Studies, Humboldt University of Berlin, Unter den Linden 6, 10099 Berlin, Germany

**Keywords:** Conditional connectives, Biconditional, Modus ponens, Affirmation of the Consequent, Denial of the Antecedent, German

## Abstract

In this paper, the meaning and processing of the German conditional connectives (CCs) such as *wenn* ‘if’ and *nur wenn* ‘only if’ are investigated. In Experiment 1, participants read short scenarios containing a conditional sentence (i.e., If P, Q.) with *wenn/nur wenn* ‘if/only if’ and a confirmed or negated antecedent (i.e., P/not-P), and subsequently completed the final sentence about Q (with or without negation). In Experiment 2, participants rated the truth or falsity of the consequent Q after reading a conditional sentence with *wenn* or *nur wenn* and a confirmed or negated antecedent (i.e., If P, Q. P/not-P. // Therefore, Q?). Both experiments showed that neither *wenn* nor *nur wenn* were interpreted as biconditional CCs. Modus Ponens (If P, Q. P. // Therefore, Q) was validated for *wenn*, whereas it was not validated in the case of *nur wenn*. While Denial of the Antecedent (If P, Q. not-P. // Therefore, not-Q.) was validated in the case of *nur wenn*, it was not validated for *wenn*. The same method was used to test *wenn* vs. *unter der Bedingung, dass* ‘on condition that’ in Experiment 3, and *wenn* vs. *vorausgesetzt, dass* ‘provided that’ in Experiment 4. Experiment 5, using Affirmation of the Consequent (If P, Q. Q. // Therefore, P.) to test *wenn* vs. *nur wenn* replicated the results of Experiment 2. Taken together, the results show that in German, *unter der Bedingung, dass* is the most likely candidate of biconditional CCs whereas all others are not biconditional. The findings, in particular of *nur wenn* not being semantically biconditional, are discussed based on available formal analyses of conditionals.

## Introduction

Conditionals (e.g., *If it rains, the streets get wet.*) are one of the most studied topics in cognitive science. Despite the vast body of literature in linguistics, logic, philosophy and psychology, there are many open questions left. This paper deals with the interpretation of conditionals in the form of e.g., “If P, Q” in natural language, with a focus on the effect of conditional connectives (CCs) such as *if* or related expressions in German, see (1).

1. German CCs:
  - Simple CCs (i.e., those that convey the antecedent proposition P as a sufficient but not necessary condition for the consequent proposition Q): *wenn* ‘when, if’, *falls* ‘if, in case’, *insofern/sofern* ‘if, in case’, *angenommen, dass* ‘assuming that’, *im Falle, dass* ‘in the event that’, *unter der Annahme, dass* ‘assuming that’
  - Arguably biconditional CCs (i.e., those that might convey P as a sufficient and necessary condition for Q): *vorausgesetzt, dass* ‘provided that’, *unter der Voraussetzung, dass* ‘provided that’, *unter der Bedingung, dass* ‘on condition that’, *nur wenn* ‘only if’

These CCs differ in syntactic properties in that some contain single connectives and some are verbal or prepositional phrases. In the current paper, we will deal with their semantic and pragmatic contributions, focusing on the question of whether they express semantic biconditionals or not. Being conditional connectives, they bear some relation to the logical connective of material implication or conditional *if* ( →) and the logical connective of material biconditional *iff* (if and only if, ↔) in first-order logic.

For material conditionals (i.e., *if-*sentences), there are two well-known valid rules of inference, with a rule of inference being valid if and only if it is logically impossible for the conclusion to be false with its premises being true. The first valid rule of inference is called Modus Ponens (MP), as stated and illustrated in (2a). The second valid rule of inference is called Modus Tollens, as stated and illustrated in (2b). On the other hand, there are two well-known invalid (fallacious) rules of inference for material conditionals, namely, Affirmation of the Consequent (AC), as in (2c), and Denial of the Antecedent (DA), as in (2d). This means, for *if*, “If P, Q” together with P entails Q, but “If P, Q” together with Q does not entail P and “If P, Q” together with not-P does not entail not-Q.

While MP, AC and DA are valid or invalid rules of inference for *if-*conditionals, they can all be indirectly validated in classical propositional logic for biconditionals (i.e., *iff*). For instance, if we treat *iff* as a conjunction of *if*s (e.g., *Iff it rains, the streets get wet* = *If it rains, the streets get wet* and *If the streets get wet, it rains.*), then MP and AC follow from conjunction-elimination in a standard natural deduction calculus for classical propositional logic. DA can be proven similarly, using *reductio ad absurdum*. Thus, in the case of *Iff it rains, the streets get wet*, where the antecedent is a necessary and sufficient condition for the consequent, we can conclude that if it does not rain, the streets do not get wet. This does not hold in the case of *If it rains, the streets get wet*, as in this case rain is a sufficient but not necessary condition, that is, if it does not rain, the streets can nevertheless get wet for other reasons, e.g., by snow.

(2)
  - Modus Ponens: valid
    If it rains, the streets get wet.: (If P, Q.)
    It rains.: (P.)
    Therefore, the streets get wet.: (Therefore, Q.)
  - Modus Tollens: valid
    If it rains, the streets get wet.: (If P, Q.)
    The streets do not get wet.: (not-Q.)
    Therefore, it does not rain.: (Therefore, not-P.)
  - Affirmation of the Consequent: invalid
    If it rains, the streets get wet.: (If P, Q.)
    The streets get wet.: (Q.)
    It rains.: (P.)
  - Denial of the Antecedent: invalid
    If it rains, the streets get wet.: (If P, Q.)
    It does not rain.: (not-P.)
    Therefore, the streets do not get wet.: (Therefore, not-Q.)

However, the treatment of natural language *if* as a truth-functional connective has received criticisms from researchers in psychology, see Evans and Over’s (2004) suppositional theory and Johnson-Laird and Byrne’s (2002) mental model theory of conditionals. Also in linguistics and philosophy, most researchers take the truth-functional analysis of conditionals to be inadequate for capturing the interpretation of various conditional sentences in natural language (e.g., von Fintel, 2011 and related work). Kratzer (1986, 1991) proposes a very influential formal semantic analysis of conditionals, the restrictor analysis, with the following essential components. First, *if* in natural language has no conditional meaning but is a restrictor that introduces the restriction in the *if-*clause. Second, a sentence such as (3) involves an overt or covert necessity modal verb (e.g., *must)* that takes semantic scope over the sentence, see (3a). Third, due to the necessity modal verb, the logical form of the sentence involves universal quantification over the set of P-worlds, i.e., the set of worlds epistemically accessible from the world of the utterance. The formal semantics of conditionals and modal verbs have been under constant revision. For the current purpose, it suffices to know that the interpretation of (3) can be paraphrased in (3b), which involves a universal quantificational analysis of bare conditionals (see Lewis’, 1918 strict conditional analysis). For bare conditionals, a valid inference is stated in (3c) and invalid inferences are for example, those in (3d) or (3e), because the streets can be wet for reasons unrelated to rain.

(3) * If it rains, the streets (must) get wet.*
  - Logical form of (3): Must(P, Q), i.e., all P-worlds are Q-worlds.
  - Paraphrase of (3): All worlds where it rains are worlds where the streets get wet.
  - Valid inference: All worlds where the streets do not get wet are worlds where it does not rain.
  - Invalid inference: All worlds where the streets get wet are worlds where it rains.
  - Invalid inference: All worlds where it does not rain are worlds where the streets do not get wet.

Follow-up formal studies of conditionals show that their interpretation is subject to semantic and pragmatic modulations, such as through mood choice, particle use, and discourse contexts (e.g., Arregui, 2005; Grosz, 2012; von Fintel, 2011). However, the role that CCs play in the interpretation of conditionals remains far from clear. It is reasonable to assume that the CCs in (1a) and (1b) do not have identical meanings. On the one hand, the notion of “conditionals” usually applies to *if-*sentences only, not to sentences with ‘only if’ or ‘if and only if’. On the other hand, they all express the antecedent as the (necessary or sufficient) condition for the consequent, and thus are related to one another. One of the central questions concerns the compositional meaning of less typical CCs. In this paper, we will report a study focusing on the semantics of the CC *nur wenn* ‘only if’ (as well as *unter der Bedingung, dass* ‘on condition that’ and *vorausgesetzt, dass* ‘provided that’) in relation to the more canonical and most frequently used CC *wenn* in German.

### From *if *to *iff* (Conditional Perfection)

While some natural language CCs are arguably inherently biconditional, for example, *if and only if* and, possibly, also *on condition that* (e.g., Montolío, 2000; Liu, 2019a), some CCs are arguably only pragmatically biconditional. Using example (4), Geis and Zwicky (1971) claim that upon hearing the sentence (4a), the hearer can additionally infer (4b), whereby the semantically weaker conditional sentence (4a) is ‘perfected’ to a semantically stronger biconditional one as in (4c).

(4)
  - *If you mow the lawn, I will give you 5 dollars.*
  - *If you don’t mow the lawn, I won’t give you 5 dollars.*
  - *If and only if you mow the lawn, I will give you 5 dollars.*

Some researchers relate conditional perfection to cases of pragmatic enrichment, e.g., as a Gricean conversational implicature (cf. van der Auwera 1997; Horn, 2000 for their different takes on this). Others take a different stance. Noveck et al. (2011), for example, argue against the pragmatic approach from a developmental and a processing perspective. One of their main arguments is that if conditional perfection is indeed a case of pragmatic enrichment, it should be linked to extra effort (i.e., with age or extra processing time), which is contrary to the existing literature: Bonnefond et al. (2012), for instance, report on a self-paced task and an EEG (electroencephalography) study on AC. AC is an invalid rule of inference (i.e., to be rejected) but should be accepted on pragmatic grounds due to conditional perfection (i.e., to be endorsed). Their results show that AC-rejecters took longer than AC-endorsers and that both rejecters and endorsers of AC produced an N200 wave that is associated with a violation of expectations, which casts doubt on the pragmatic accounts of conditional perfection. Thus, how logical fallacies of AC or DA relate to conditional perfection including the aspect of individual variation remains to be further investigated. Furthermore, Van Canegem-Ardijns and van Belle (2008) point out that conditionals differ in the cancellability of the invited inference, as illustrated by their examples in (5): the cancellation of the invited inference is odd in (5a) but fine in (5b).

(5)
  - * If you mow the lawn, I will give you 5 dollars. #But if you don’t mow the lawn, I will give you 5 dollars anyway.*
  - *If the weather is good tomorrow, I’ll go for a swim. But if the weather is not good tomorrow, maybe I’ll go for a swim anyway.*

This shows that whether a bare conditional is perfected to a biconditional is subject to contextual manipulations, which raises additional questions about the semantic or pragmatic status of the invited inference. While the contrast in the minimal pair in (5) is due to the specific antecedent/consequent relation and world knowledge, we will look into the exact influence of the type of CCs and variation among them in this regard, focusing on the German CCs *nur wenn* (‘only if’) vs. *wenn* (‘if/when’).

### From *if* to *only if*

In order to discuss the meaning of *nur wenn* ‘only if’, we need to briefly present our assumptions for the focus particle *nur* ‘only’. Following Horn (2002), an *only-*sentence expresses two entailments, as illustrated in (6).

(6) *Only Peter came.*
  - **entailment/assertion:** Nobody other than Peter came.
  - **entailment/assertoric inertia:** Peter came.

Crucially, these two entailments have different pragmatic statuses, as the negative entailment (6a) is asserted and the positive one (6b) is “assertorially inert” (treated as a presupposition in Horn, 1989). Horn uses this asymmetry to explain why *only-*sentences can license negative polarity items (NPIs), i.e., expressions that need licensing by negative contexts such as *any* and *ever* (Ladusaw, 1980; von Fintel, 1999). Namely, it is negative at the assertion level where NPIs are licensed. Taking from there, we can derive the meaning of *only if* or *nur wenn* in German as in (7): by the positive entailment, “Only if P, Q” together with P entails Q, and by the negative entailment, “Only if P, Q” together with not-P entails not-Q. Crucially, due to the two entailments, *only if* seems to be biconditional. That is, P is not only the sufficient condition for Q (as in bare conditionals) but also the necessary condition for Q. Combined with Kratzer’s universal quantificational analysis of conditionals in (3), the sentence would involve universal quantification at both propositions, as indicated in (7a) and (7b).

(7) *Only if it rains, the streets get wet.* (Only if P, Q.)
  - **entailment/assertion:**    If not-P, not-Q.
    i.e., all worlds where it does not rain are worlds where the streets do not get wet.
  - **entailment/assertoric inertia:**      If P, Q.
     i.e., all worlds where it rains are worlds where the streets are wet.

The question of whether this is the right take on the semantics of *nur wenn* or its correlates needs to be discussed with reference to *conditional perfection*, which we introduced above. Whereas conditional perfection concerns the inference of “If not-P, not-Q.” in *if-*sentences, some researchers have also raised questions about the inference “If P, Q” in the case of *only-if-*sentences. Using the example in (8), Herburger (2015, 2019) argues that *only-if-*sentences (or conditionals under *only*/negative/downward-entailing contexts) do not presuppose that “all (normal) instances of hard work will be rewarded by success” – that is, (8) does not presuppose or entail (8b), unlike in the case of bare conditionals such as *If you work hard, you succeed.* In her words, bare conditionals exhibit “Conditional Duality” as they involve different quantificational force: “In upward entailing contexts we find the universal reading, in downward entailing contexts the existential one.” (Herburger, 2019: 143).

(8) *Only if you work hard do you succeed.* (only [if P, Q])
  - **Universal conditional: for bare *if*-conditionals**
    In certain contexts, [If P, Q] is true iff all P-cases are Q-cases.
  - **Existential conditional: for *if*-conditionals under *only***
    In certain contexts, [If P, Q] is true iff some P-cases are Q-cases.

(8) Is used as a motivation for people to work hard. This pragmatic function is achieved through the combination of the two inferences with different quantificational force, namely, some P-cases are Q-cases (i.e., some hard work results in success) and all not-P-cases are not-Q-cases (i.e., laziness results in lack of success). While Herburger’s (2015, 2019) analysis of *only if* is based on introspective data and formal reasoning, we will report experimental data that provide convergent supporting evidence.

### Scope and Structure of the Paper

As the first step, we searched for the occurrences of the three CCs *wenn/nur wenn/wenn und nur wenn* ‘if/only if/if and only if’ in the German Reference Corpus (DeReKo, IDS (2020a), accessed 09/2020) with over 46.9 billion words. We found over 10.000.000 instances of *wenn* ‘if/when’ and 193.815 instances of *nur wenn* ‘only if’. In contrast, we found only 35 occurrences of *wenn und nur wenn* ‘if and only if’. This shows that the biconditional CC is rarely used in natural language, which is echoed in one of the found examples: *“wenn und nur wenn” ist nur im Englischen gebräuchlich, nicht jedoch im Deutschen* (“if and only if” is only used in English but not in German). It is to note, though, that in formal logic, *genau dann, wenn* is used in German as the counterpart of *if and only if.*

In the following, we report on our investigations of German CCs with regard to their semantic biconditionality, with a focus on *wenn* ‘if’, *nur wenn* ‘only if’, *wenn und nur wenn* ‘if and only if’ as well as *unter der Bedingung, dass* ‘on condition that’ and *vorausgesetzt, dass* ‘provided that’. Using corpus- and psycholinguistic methods, we take MP and DA/AC inferences as criteria, with the following questions in (9). For a specific CC, if the answer is yes to both the MP and the DA (or alternatively, to both the MP and the AC) question, we take it to be semantically biconditional. Otherwise, it is not biconditional.

(9) Questions to be tested for a CC:
  - MP-inference**:** Does “CC P, Q” together with P entail Q?
  - DA-inference: Does “CC P, Q” together with not-P entail not-Q?
  - AC-inference: Does “CC P, Q” together with Q entail P?

The paper is organized as follows. In Sect. 2, we report the results of a written sentence production task. Based on the collected data, we conducted qualitative analyses of each CC with regard to the MP and the DA inferences. The results show that *wenn und nur wenn* is biconditional, whereas *wenn* and *nur wenn* are not. In Sect. 3, we report a written sentence completion experiment (Experiment 1) in which participants completed conditional scenarios. In Sects. 4 to 7, we present four sentence rating experiments (Experiments 2–5) in which participants rated inferences of conditionals using *nur wenn* vs. *wenn* (Experiment 2 and 5)*, vorausgesetzt, dass* vs. *wenn* (Experiment 3) and *unter der Bedingung, dass* vs. *wenn* (Experiment 4). We discuss the results and conclude in Sect. 8.

## Written Sentence Production Task

Due to scarce occurrences of *wenn und nur wenn* in the corpus, we conducted a sentence completion study with a qualitative analysis to get a balanced data set of the three CCs. However, the choice of *wenn und nur wenn* as the biconditional CC candidate in German might be problematic, as evidenced by the low count in the corpus; we will get back to this point in Sect. 8. We also collected data with the CC *falls* for a different study in relation to Liu (2019b, 2021) but will not discuss them here.

### Method

#### Participants

One hundred German native speaking participants (37 female, 1 non-binary, mean age = 27, SD = 7.8) participated in the study through the online crowd-sourcing platform Prolific (https://www.prolific.co/). They were compensated with small payments.

#### Materials

The study comprised three sub-tasks presented one after another: familiarity judgments for various German expressions, a sentence completion task, and an author recognition test (ART). The first and the last sub-tasks were used for a different study relating to degree modifiers in German. They bear no relation to CCs, and thus will not be analyzed or discussed here.

For the sentence completion task, two types of sentence fragments were presented. In the first type, participants saw CCs in sentence-initial position. Each sentence contained one of the CCs *Wenn ‘if’, Nur wenn* ‘only if’, or *Wenn und nur wenn* ‘if and only if’. Participants were asked to complete the sentence however they liked but grammatically correct. In the second type, participants saw a sentence-initial name or pronoun, e.g., *"Ich..." *(‘I...’) and were asked to complete the sentence using one of the three CCs. We also included 8 filler fragments, for which participants were asked to complete the sentence using the CC *falls* or one of the degree modifiers, i.e., *sonderlich* ‘all that’, *sehr* ‘very’, *so recht* ‘really’, either sentence-initially (4 out of 8) or not (4).

#### Procedure

The experiment was implemented on Ibex Farm (Drummond, 2013). The three sub-tasks were presented in the following order: familiarity judgments, first half of the production task, ART, second half of the production task. In the production task, sentence fragments were presented in a randomized order. They showed up in the middle of the screen together with a text box where the participants were asked to type their continuation for the sentence. The experiment took around 15 min in total.

### Data Analysis and Results

We received 200 sentences for each of the three CCs occurring either sentence-initially (100) or not (100). We conducted qualitative analyses of the produced sentences regarding their biconditionality. First, we removed irrelevant sentences, e.g., *Wenn es wirklich sein muss* ‘If it really has to be’, that is, sentences without consequents. Next, a research assistant coded whether MP and DA inferences were valid for each of the sentences. To check biconditionality, we started with DA instead of AC inferences, as DA (i.e., If P, Q. not-P. // Therefore, not-Q; see (2d)) reserves the temporal or causal relation between the antecedent and the consequent proposition in the given conditionals, but we will show later with Experiment 2 and 5 that the experimental results based on them are comparable, at least in the current study.

As shown in Table 1, both MP inferences (i.e., whether “CC P, Q” with P entails Q) and DA inferences (i.e., whether “CC P, Q” with not-P entails not-Q) were judged as valid for all the relevant sentences with *wenn und nur wenn,* confirming that it is a biconditional connective. Different patterns emerge for both w*enn* and *nur wenn*, though: The MP inference was judged to be valid for almost all *wenn-*sentences but invalid for 9.2% of the relevant *nur-wenn-*sentences. The DA inference, on the other hand, was judged to be valid for almost all *nur-wenn-*sentences, but only for roughly two-thirds of the *wenn-*sentences.

**Table 1:** Subjective MP and DA judgment data of the written sentence production study

CC	Modus Ponens		Denial of the Antecedent		Irrelevant
	valid	invalid	valid	invalid	
*wenn*	180 (99.4%)	1 (0.6%)	121 (66.9%)	60 (33.1%)	19
*nur wenn*	167 (90.8%)	17 (9.2%)	180 (97.8%)	4 (2.2%)	16
*wenn und nur wenn*	183 (100%)	0 (0)	183 (100%)	0 (0%)	17

### Discussion

The sentence completions provided by participants were annotated with regard to the validity of the MP/DA inference (see (9) above). The results revealed that *wenn und nur wenn,* while being rarely used in German, is a biconditional connective. For *wenn*, the MP inference (e.g., *Wenn ich Hunger habe, esse ich. Ich habe Hunger. // Ich esse.* ‘If I am hungry, I will eat. I am hungry. *//* I will eat.’) was judged as valid*,* while the DA inference (e.g., *Wenn ich Hunger habe, esse ich. Ich habe keinen Hunger. // Ich esse nicht.* ‘If I am hungry, I will eat. I am not hungry. *//* I will not eat.’) was not consistently judged as valid*.* This is in line with the literature stating that bare conditional sentences entail that all P-worlds are Q-worlds (Kratzer, 1986/1991, Lewis, 1918), and that they give rise to conditional perfection only in some cases (Geis & Zwicky, 1971; Van Canegem-Ardijns & van Belle, 2008). In the case of *nur wenn,* the DA inference (e.g., *Ich weine nur wenn ich traurig bin. Ich bin nicht traurig. // Ich weine nicht.* ‘Only if I am sad will I cry. I am not sad. // I will not cry.’) was unsurprisingly judged as valid, see (7a). However, the MP inference (e.g., *Ich weine nur wenn ich traurig bin. Ich bin traurig. // Ich weine.* ‘Only if I am sad will I cry. I am sad. // I will cry.’) was only judged as valid in some cases, casting doubt on the analysis in (7b) and providing tentative evidence for Herburger’s (2015/2019) analysis of “*Only if you work hard do you succeed.*” in (8b). While this study provides a first step to understand the differences and similarities between these CCs, the annotator’s judgements are inconclusive due to their subjective nature. Thus, we conducted four additional experiments using different designs and measures with a focus on *wenn* versus *nur wenn*, which we will present in the following sections.

## Experiment 1

In this experiment, we used a sentence completion task to tackle the meaning difference between *wenn* and *nur wenn*.

### Method

#### Participants

Eighteen German native speakers (8 female, mean age = 30, SD = 8.6) participated in the study online via Prolific and were compensated with small payments. None of the participants took part in more than one of the following experiments.

#### Materials and Design

108 scenarios were constructed with 4 sentences each (Sentence1-4, see (10)), based on a 2 × 2 (CC × Antecedent) factorial design. Sentence1 (S1) set the scenario context. In the two critical conditions, S2 was a conditional sentence, containing either the CC *wenn* or *nur wenn*. S3 either confirmed or falsified the antecedent in S2 (P/not-P). S4 presented the beginning of a sentence containing the consequent in S2 and had to be completed by participants. Notably, S4 could be completed by either confirming or negating the consequent (e.g., *Von denen schnitt er (k)einen aus.* ‘From those he cut one/none out’). In an additional filler condition, S2 was not a conditional sentence and instead contained the possibility modal adverb *vielleicht* (e.g., *Vielleicht schneide ich einen aus.* ‘Maybe I cut one out’).

(10)
  S1: *Kristian las die Zeitung und dachte sich:*
    (Kristian read the newspaper and thought:)
  S2: *Wenn / Nur wenn die Artikel interessant sind, schneide ich einen aus.*
    (If / Only if the articles are interesting, I’ll cut one out.)
  S3: *Wie sich zeigte, waren die Artikel (nicht) interessant.*
    (As became apparent, the articles were (not) interesting.)
  S4: *Von denen schnitt er…*
    (Of those he cut…)

We assume that MP inferences are valid for *wenn*-conditionals but invalid for *nur-wenn*-conditionals, whereas DA inferences are valid for *nur-wenn*-conditionals, but invalid for *wenn*-conditionals, see Sects. 1, 2. This leads to the prediction of an interaction between CC and Antecedent: After negated antecedents (i.e., “CC P, Q” with not-P), more negative consequents (not-Q) are predicted for *nur wenn* than for *wenn*; after positive antecedents (i.e., “CC P, Q” with P), more positive consequents (Q) are predicted for *wenn* than for *nur wenn*.

#### Procedure

The experiment was implemented on Ibex Farm (Drummond, 2013). Each trial began with a fixation cross for one second that was replaced by S1. Participants pressed the space bar at their own pace to replace each of the scenario sentences with the next one until reaching S4, which they had to complete based on the scenario in S1-S3. Four counter-balanced experimental lists were designed with equal numbers of trials per condition in each of the lists, so that each item would be presented in only one of the conditions per list. Participants were randomly assigned to one of the lists. The whole test took around 20 min.

### Results

**Fig. 1:**
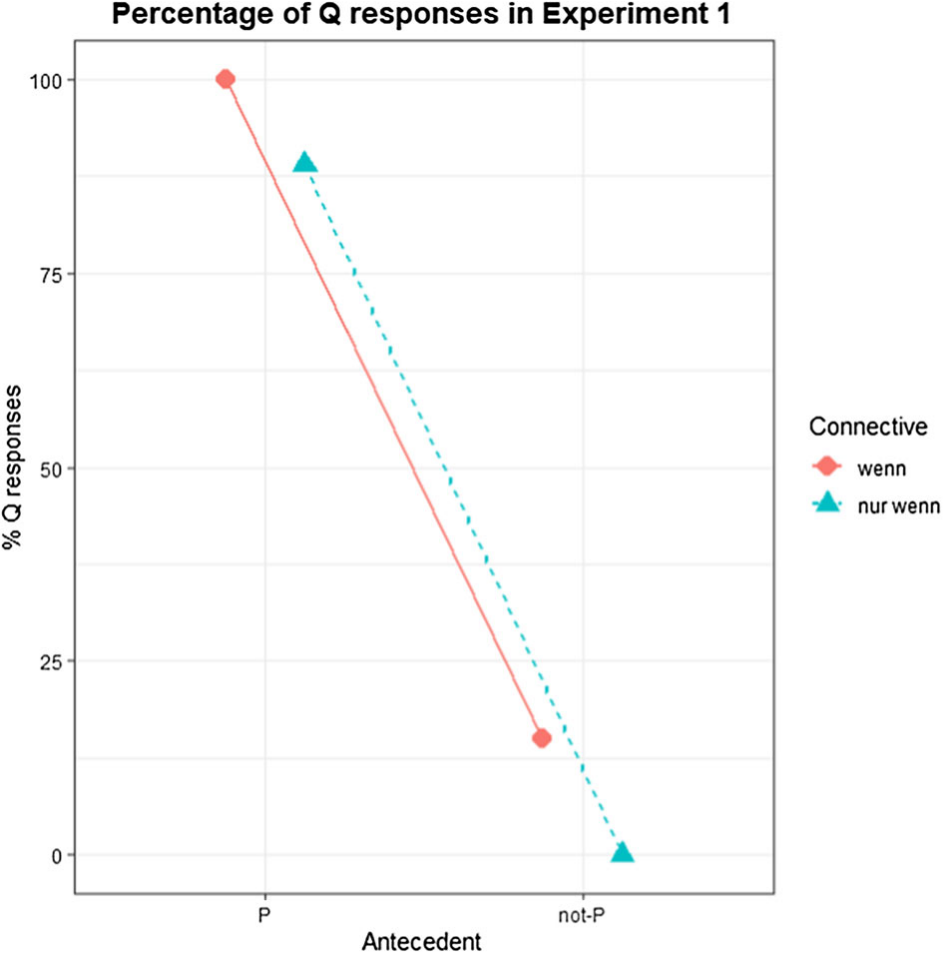
Percentages of positive consequent (Q) responses by condition in Experiment 1

Sentence completion responses were categorized as ‘negative consequent’ (not-Q) when the consequent contained negative or downward entailing (Ladusaw, 1980) expressions such as the quantifier *kein* ‘no’ in the response; otherwise, they were categorized as ‘positive consequent’ (Q), see Fig. 1. A Bayesian multinomial regression model was fitted using *brms* (Bürkner, 2017) with the factor CC (*wenn/nur wenn,* or without CC) and the factor Antecedent (P/not-P) as well as their interaction as fixed effects and as random effects by subject and by item. Both CC and antecedent were dummy coded, with *wenn* and ‘not-P’ as reference levels. Responses were coded as Q = 1 and not-Q = 0. Table 2 shows the model output. For the interpretation of Bayes factors and effect strengths, we follow the labeling proposed by Andraszewicz et al. (2015), see also Lee and Wagenmakers (2014).

**Table 2:** Bayesian regression model output on sentence completions in Experiment 1

	Estimate	l-95% CI	u-95% CI
*Intercept*	− 2.57	− 3.53	− 1.74
*CCnur_wenn*	− 0.64	− 1.57	0.12
*CCnon_cond*	1.52	0.49	2.56
*Antecedent_pos*	11.82	8.42	16.88
*CCnur_wenn:Antecedent_pos*	5.15	− 1.97	17.87
*CCnon_cond:Antecedent_pos*	− 4.59	− 9.16	0.10

Formula = responseCategory ~ 1 + CC * Antecedent + (1 + CC * Antecedent|subjectID) + (1 + CC * Antecedent|itemID); Family = bernoulli

The *hypothesis* function implemented in *brms* was used to test whether more negative consequent (not-Q) responses were given in *nur wenn* than in *wenn* after negated antecedents (not-P), which was confirmed (*β* = − 0.64; CI = [− 1.4; 0]; BF_10_ = 19), with 99% for *nur wenn* and 85% for *wenn*. After positive antecedents (P), decisively more positive consequent (Q) responses were produced than after negative antecedents (not-P) in *wenn* (*β* = 11.82; CI = [8.84; 15.85]; BF_10_ > 6000). This effect is moderately bigger in *nur wenn* (*β* = 5.15; CI = [− 0.92; 14.71]; BF_10_ = 9), as indicated by the interaction effect of CC and Antecedent. After positive antecedents (P), more positive consequents were produced after *wenn* (100%) than after *nur wenn* (89%), with the respective effect in the model being of moderate size (*β* = 4.51; CI = [− 1.53; 14]; BF_10_ = 6).

### Discussion

Experiment 1 tested participants’ preferences for scenario completions with conditional sentences containing positive or negated antecedents and different CCs.

Negated antecedents generally led to increased numbers of negated consequents in the completions. While this trend was almost perfect in the *nur wenn* condition, 15% of completions contained positive consequents in the *wenn* condition. This indicates that *nur wenn* triggers the not-P → not-Q inference in all cases (i.e., “Nur wenn P, Q.” together with not-P entails not-Q, or to put differently, the DA inference can be validated), in line with the analysis in (7), which takes it as one of the semantic entailments of the sentence. The result about *wenn* shows that the not-P → not-Q inference is not part of its semantics but a pragmatic inference. The high rate of negative consequents can be explained in relation to conditional perfection.

Positive antecedents were generally found to lead to positive consequents in the scenario completions. In this case, the trend was almost absolute in *wenn*, while 11% of completions contained negative consequents in *nur wenn*, indicating that MP is valid in *wenn* but not necessarily so in *nur wenn*. This finding is surprising, given the analysis of *nur wenn* in (7), but in line with the analysis of Herburger (2015, 2019) as well as our MP judgments for it in the Written Sentence Production Task (Sect. 2). Taken together, both studies show that neither *wenn* nor *nur wenn* are biconditional and that they differ in both the MP and the DA inferences. As both studies involve production data, we conducted two sentence rating experiments to investigate the meaning of *nur wenn* vs. *wenn* further (Experiment 2 and 5), which we are going to present in the following sections.

## Experiment 2

The results of the sentence completion experiment above hinted at a meaning difference between the CCs *wenn* and *nur wenn*. In this experiment, this difference will be tested on similar scenarios with a more sensitive measure, that is, sentence ratings.

### Method

#### Participants

Forty-eight German native speakers (21 female, 1 non-binary, mean age = 29, SD = 8.6) were recruited as paid participants online via Prolific.

#### Materials and design

Forty-eight critical items were composed as sets of three sentences each, see (11). Sentence 1 (S1) contained a conditional of the format ‘CC P, Q.’, using either the CC *wenn* or *nur wenn*. S2 presented either the antecedent proposition P or not-P in isolation. S3 asked for the validity of the consequent Q. Hence, the experiment comprised a 2 × 2 design, with CC (*wenn* vs. *nur wenn*) and Antecedent (P vs. not-P) as factors. Participants were asked to rate a set of sentences presenting an inference based on MP (S1: If P, Q. S2: P. S3: Q?) or DA (S1: If P, Q. S2: not-P. S3: Q?).

(11)
  S1: *Wenn / Nur wenn heute gutes Wetter ist, geht Kai Eis essen.*
    (If / Only if the weather is nice today, Kai will go have ice cream.)
  S2: *Heute ist gutes* Wetter. / *Heute ist kein gutes Wetter.*
    (The weather is nice today. / The weather is not nice today.)
  S3: *Geht Kai Eis essen?* (Will Kai go have ice cream?)

Additionally, 48 filler items were tested, using different (non-conditional) connectives such as *entweder—oder* (either or), *oder* (or), *und* (and), *weil* (because), and *aber* (but). The amount of MP-like and DA-like fillers was counter-balanced. 8 practice trails mimicking critical items and fillers preceded the experiment. Four counter-balanced experimental lists were designed with equal numbers of trials per condition in each of the lists, so that each item would be presented in only one of the conditions per list. Participants were randomly assigned to one of the lists.

Based on the theoretical assumptions in Sect. 1 and the empirical findings in Sects. 2, 3, we assume that MP inferences are valid for *wenn*-conditionals but invalid for *nur-wenn*-conditionals, whereas DA inferences are valid for *nur-wenn*-conditionals, but invalid for *wenn*-conditionals. This leads to the prediction of an interaction between CC and Antecedent: After negated antecedents (i.e., “CC P, Q” with not-P), lower ratings to Q are predicted for *nur wenn* than for *wenn*; after positive antecedents (i.e., “CC P, Q” with P), higher ratings to Q are predicted for *wenn* than for *nur wenn*. That is, we expect higher ratings for *wenn* than for *nur wenn* in both MP and DA conditions.

Furthermore, we also measure reaction latencies with no specific predictions. We will report on these, but will not base our conclusions on them.

#### Procedure

The experiment was implemented on Ibex Farm (Drummond, 2013). Each trial began with a fixation cross in the middle of the sentence to appear and each of the sentences replaced the preceding sentence in the same position. Participants were instructed to read each of the three sentences in each trial carefully at their own pace and press the space bar whenever they were ready to see the next sentence. They were further instructed to quickly answer the final question in S3 based on their intuition by clicking on one of the five answers that were presented below S3: *Nein.* (no), *Eher nein.* (rather no), *Das ist nicht sicher.* (that is not certain), *Eher ja.* (rather yes), and *Ja.* (yes). The whole testing session took about 30 min.

### Results

**Fig. 2:**
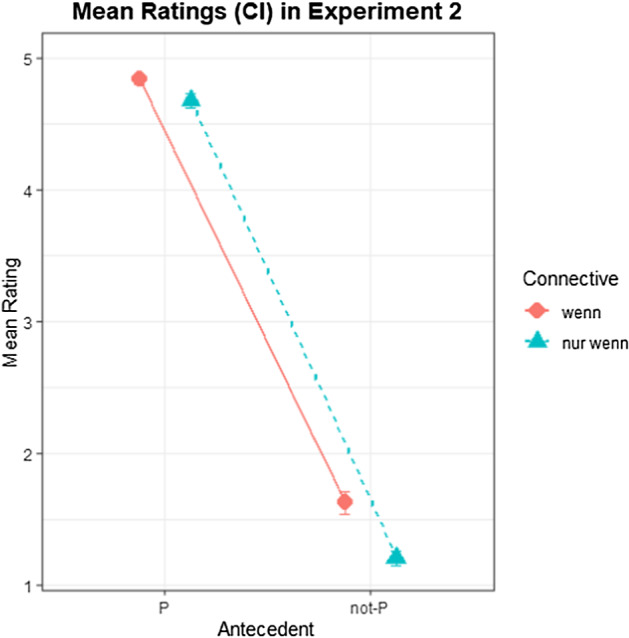
Mean ratings for Q (1 = no, 5 = yes) by condition in Experiment 2. Error bars indicate 90% CI.

Data from two of the 48 participants were excluded due to random response patterns, especially in filler trials that were not questionable, such as for instance coordinated clauses (e.g., “Marco ruft beim Kino an und reserviert Karten für die Abendvorstellung.” // “Marco reserviert Karten für die Abendvorstellung.” // “Ruft er beim Kino an?” (M. calls the cinema and reserves tickets. // M. reserves tickets. // Does he call the cinema?)). Of the 2208 trials, 8 were discarded because ratings were not given within 10 s. Another 48 trials were discarded because their response time deviated more than 3 SDs (standard deviations) from their respective subject means. Participant ratings by condition are shown in Fig. 2. A Bayesian ordinal regression model was fitted with CC, Antecedent and their interaction as fixed effects as well as random effects by subject and by item. Both CC and Antecedent were dummy coded, with *wenn* and P as reference levels. Table 3 shows the model output.

**Table 3:** Bayesian regression model output on sentence ratings in Experiment 2

	Estimate	I-95% CI	u-95% CI
*Intercept[1]*	− 8.43	− 9.38	− 7.64
*Intercept[2]*	− 6.40	− 7.29	− 5.64
*Intercept[3]*	− 4.93	− 5.78	− 4.21
*Intercept[4]*	− 2.95	− 3.74	− 2.29
*CC_nurwenn*	− 1.09	− 1.86	− 0.36
*Antecedent_not-P*	− 9.21	− 10.48	− 8.08
*CC_nurwenn:Antecedent_not-P*	− 0.99	− 1.82	− 0.24

Population-Level Effects. Formula = response_ord ~ 1 + CC * Antecedent + (1 + CC * Antecedent|subjectID) + (1 + CC * Antecedent|itemID); Family = cumulative

Hypothesis-specific tests were conducted with *brms* and showed a decisive main effect of Antecedent, with lower ratings for Q in DA than in MP (*β* = − 19.4; CI = [− 21.35; − 17.62]; BF_10_ > 3000). Moreover, they showed a decisive main effect of CC, with ratings for *wenn* being higher than for *nur wenn* (*β* = − 3.18; CI = [− 4.32; − 2.11]; BF_10_ > 3000). Further, they showed a clear interaction effect of CC with Antecedent, with the effect of Antecedent being slightly stronger in *nur wenn* than in *wenn* (*β* = − 0.99; CI = [− 0.39; − 1.63]; BF_10_ = 175). Crucially, ratings were higher in *wenn* than in *nur wenn* in both MP (*β* = − 1.09; CI = [− 1.72; − 0.49]; BF_10_ = 499) and DA (*β* = − 2.08; CI = [− 2.78; − 1.45]; BF_10_ > 3000).

**Fig. 3:**
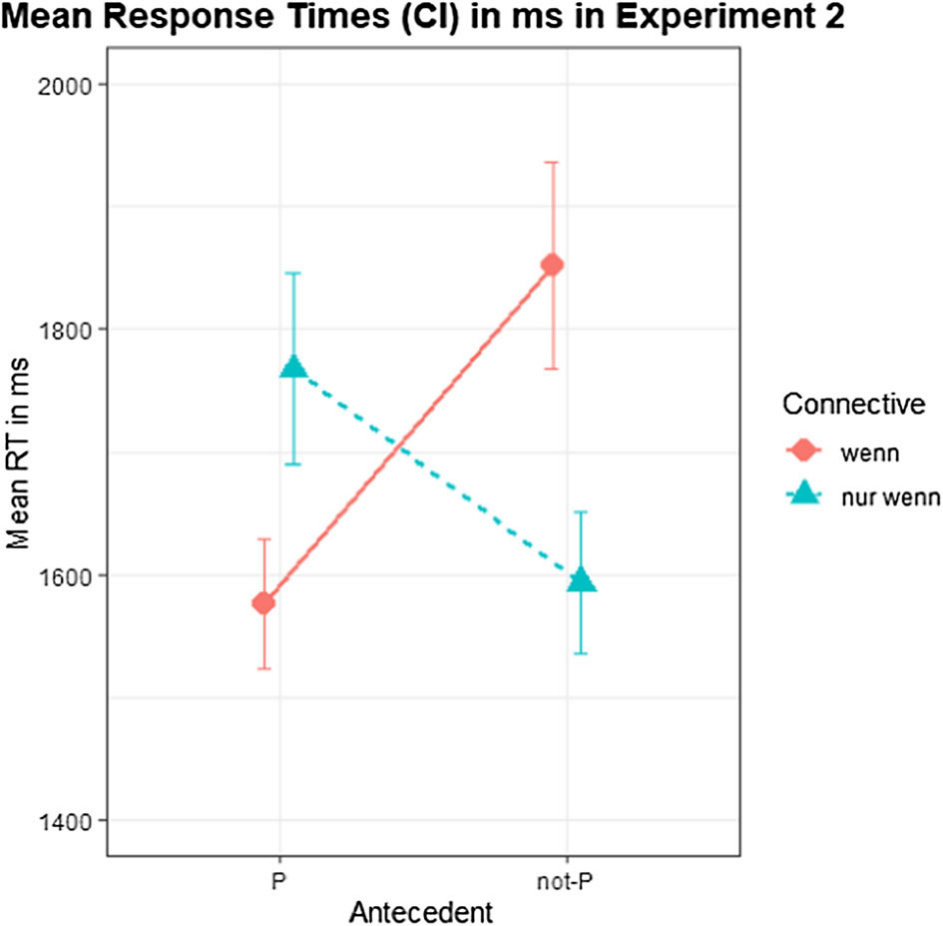
Mean decision latencies by condition in Experiment 2. Error bars indicate 90% CI.

Participants’ decision times are shown in Fig. 3. A Bayesian linear mixed-effects model was used to fit participants’ decision times using the same maximal effects structure. Table 4 shows the model output.

**Table 4:** Bayesian regression model output on rating latencies in ms in Experiment 2

	Estimate	I-95% CI	u-95% CI
*Intercept*	1571.11	1444.65	1695.27
*CC_nurwenn*	197.38	94.92	301.31
*Antecedent_not-P*	279.13	136.44	420.27
*CC_nurwenn:Antecedent_not-P*	− 458.10	− 660.65	− 254.24

Population-Level Effects. Formula = responseTime ~ 1 + CC * Antecedent + (1 + CC * Antecedent|subjectID) + (1 + CC * Antecedent|itemID); Family = gaussian

A hypothesis-specific test for an interaction of CC with Antecedent yielded decisive results, indicating a cross-over interaction (*β* = − 458.1; CI = [− 628.5; − 284.39]; BF_10_ > 3000), with reaction latencies being faster for *wenn* than for *nur wenn* in the P conditions but slower for *wenn* than for *nur wenn* in the not-P conditions. Two further hypothesis-specific tests showed weak evidence for main effects of Antecedent (*β* = 100.16; CI = [− 51.5; 250.22]; BF_10_ = 6) and CC (*β* = − 63.35; CI = [− 190.91; 64.6]; BF_10_ = 4).

### Discussion

In this experiment, we tested the validity of MP and DA arguments in conditionals containing the CCs *wenn* and *nur wenn*. The rating results indicate that MP is generally valid in *wenn*, with clear decisions for Q, but not necessarily always in *nur wenn*, which shows inconsistent decisions. Meanwhile, DA is generally valid in *nur wenn*, with clear rejections of Q, but not necessarily always in *wenn*, which does not always show clear rejections. This supports the assumption that *wenn* is a simple conditional that can get a biconditional interpretation by conditional perfection in most (but not all) cases, and that *nur wenn* is not a biconditional connective for a different reason, as MP is not equally endorsed as with *wenn*.

In the reaction latency data, we found faster reactions for acceptance of Q in the case of positive antecedents (i.e., endorsing MP) in *wenn* than in *nur wenn*, which shows slower decisions that are not always fully embracing. Meanwhile, we found faster reactions for rejections of Q in the case of negative antecedents (endorsing DA) in *nur wenn*, than in *wenn*, which shows slower decisions that are not always clear rejections. However, as we did not have clear predictions about reaction latencies, we would not base our interpretations of the data on these.

Furthermore, in this experiment’s design, we used DA along with MP as in Experiment 1 to reserve the temporal or causal relation between the antecedent (e.g., *the weather is nice today*) and the consequent proposition (e.g., *Kai will go have ice cream*) in the given conditional (e.g., *If/Only if the weather is nice today, Kai will go have ice cream*). As DA and AC inferences can be derived from each other via contraposition, the question arises whether the results can be replicated if we use AC along with MP. For this purpose, we conducted Experiment 5, to be reported in Sect. 7. Before that, Sects. 5 and 6 present two further experiments testing another two CCs for their potentially biconditional semantics: *unter der Bedingung dass* (‘on condition that’; Experiment 3) and *vorausgesetzt dass* (‘provided that’; Experiment 4).

## Experiment 3

### Method

Fifty German native speakers (23 female, 1 non-binary, mean age = 30 years, SD = 8.1) were recruited as paid participants online via Prolific. Materials and design were identical to Experiment 2, except that *nur wenn* (‘only if’) was replaced by *unter der Bedingung, dass* (‘on condition that’). The procedure was the same as in Experiment 2. For *unter der Bedingung, dass,* we examined 1) whether “CC P, Q” with P entails Q, i.e., whether it would receive high ratings for Q in the MP condition, and 2) whether “CC P, Q” with not-P entails not-Q, i.e., whether it would receive low ratings for Q in the DA condition. If both 1) and 2) hold for *unter der Bedingung, dass*, we take it to be semantically biconditional. Otherwise, it is not biconditional. We also measured the reaction latencies, with no specific predictions.

### Results

Data of two of the 50 participants were discarded because they did not do the task properly, as was apparent by their distribution of ratings, especially in the unquestionable filler trials.

**Fig. 4:**
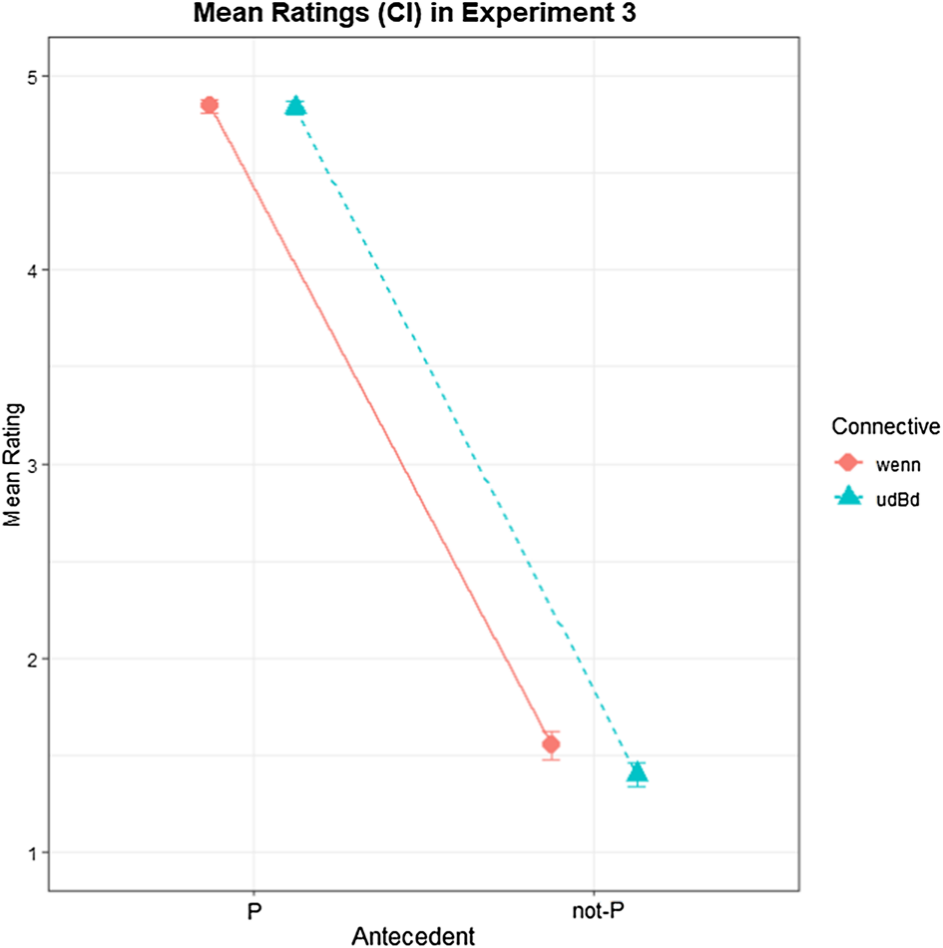
Mean ratings for Q (1 = no, 5 = yes) by condition in Experiment 3. Error bars indicate 90% CI.

Of the 2304 trials, 20 were discarded because the respective ratings were not given within 10 s. Another 48 trials were discarded because their response time deviated more than 3 SDs from their respective subject means. Participant ratings by condition are shown in Fig. 4. A Bayesian ordinal regression model on participant ratings was fitted with CC, Antecedent and their interaction as fixed effects as well as random effects by subject and by item. Both CC and Antecedent were dummy coded, with *wenn* and P as reference levels. Table 5 shows the model output.

**Table 5:** Bayesian regression model output on sentence ratings in Experiment 3

	Estimate	I-95% CI	u-95% CI
*Intercept[1]*	− 9.50	− 10.54	− 8.53
*Intercept[2]*	− 6.95	− 7.93	− 6.02
*Intercept[3]*	− 5.34	− 6.29	− 4.49
*Intercept[4]*	− 3.04	− 3.91	− 2.28
*CC_udBd*	− 0.08	− 0.60	0.45
*Antecedent_not-P*	− 10.37	− 11.79	− 9.03
*CC_udBd:Antecedent_not-P*	− 0.81	− 1.64	− 0.08

Formula = response_ord ~ 1 + CC * Antecedent + (1 + CC * Antecedent|subjectID) + (1 + CC * Antecedent|itemID); Family = cumulative

Hypothesis-specific tests conducted with *brms* (Bürkner, 2017) showed a strong interaction effect of CC with Antecedent, with the effect of CC being stronger in the not-P than in the P conditions (*β* = − 0.81; CI = [− 1.48; − 0.19]; BF_10_ = 60.22). There was a decisive main effect of Antecedent, with lower ratings for Q in the not-P (i.e., DA) conditions than in the P (i.e., MP) conditions (*β* = − 21.55; CI = [− 23.97; − 19.26]; BF_10_ > 3000). Moreover, they showed a decisive main effect of CC, with ratings for *wenn* being higher than for *unter der Bedingung, dass* (*β* = − 0.96; CI = [− 1.62; − 0.31]; BF_10_ > 3000). This main effect of CC, however, is decisive only in the not-P conditions (*β* = − 0.88; CI = [− 1.4; − 0.43]; BF_10_ = 2999) but not in the P conditions (*β* =− 0.08; CI = [− 0.51; 0.36]; BF_10_ = 1.67). These findings indicate that ratings in the P condition were statistically identical between CCs, while ratings in the not-P condition were reliably lower for *unter der Bedingung, dass* than for *wenn*.

A Bayesian linear mixed-effects regression model on participants’ decision latencies was fitted with CC, Antecedent and their interaction as fixed effects as well as random effects by subject and by item. Table 6 shows the model output. Besides a decisive main effect of Antecedent (*β* = 435.75; CI = [265.81; 611.91]; BF_10_ > 3000) indicating faster reactions in the P than in the not-P conditions, no main effect of CC (*β* = − 3.27; CI = [− 83.76; 77.45]; BF_10_ = 1.1), and no interaction of CC and Antecedent (*β* = 22.61; CI = [− 112.37; 155.57]; BF_10_ = 1.58) were found.

**Table 6:** Bayesian regression model output on rating latencies in ms in Experiment 3

	Estimate	I-95% CI	u-95% CI
*Intercept*	1652.17	1485.64	1820.38
*CC_udBd*	− 3.27	− 98.22	92.01
*Antecedent_not-P*	206.57	99.97	313.40
*CC_udBd:Antecedent_not-P*	22.61	− 139.09	183.49

Formula = responseTime ~ 1 + CC * Antecedent + (1 + CC * Antecedent|subjectID) + (1 + CC * Antecedent|itemID); Family = gaussian

### Discussion

Experiment 3 compared ratings for short natural language scenarios containing conditionals with the CCs *wenn* (‘if’) or *unter der Bedingung, dass* (‘on condition that’) that required participants to rate the validity of the MP and the DA inferences. Ratings for *wenn* were compared to ratings for *unter der Bedingung, dass* in order to test the latter for its potential biconditionality. For a CC to be semantically biconditional, both MP and DA inferences need to be rated as valid. This pattern of results was indeed found for *unter der Bedingung, dass*. As with *wenn* (or any other simple CC), ratings for *unter der Bedingung, dass* were at ceiling for MP, indicating that MP is valid for both CCs. Ratings for DA, however, were reliably lower for *unter der Bedingung, dass* than for *wenn*. While DA ratings for *wenn* were also quite low, they were decisively higher than ratings for *unter der Bedingung, dass*, indicating that DA inferences were often (but not always) acceptable also for *wenn* because of conditional perfection (a.o., van der Auwera, 1997). This pragmatic enrichment seems quite reasonable in the case of the presented scenarios, given that they presented rather minimalistic situations in which the antecedent was the only mentioned possible cause for the consequent, so that it was often interpreted to be a necessary condition for the consequent. DA ratings for *unter der Bedingung, dass*, however, were even lower, which serves as evidence for *unter der Bedingung, dass* being semantically biconditional or at least a very good candidate for a biconditional CC. Using the same method, we tested a second candidate for a biconditional CC, *vorausgesetzt, dass* (‘provided that’), in Experiment 4, which is presented in the following section.

## Experiment 4

### Method

Forty nine German native speakers (23 female; mean age = 30 years, SD = 7.7) were recruited as paid participants online via Prolific. Materials and design were identical to Experiments 2, except that *nur wenn* (‘only if’) was replaced by *vorausgesetzt, dass* (‘provided that’). The procedure was the same as in Experiment 2. For *vorausgesetzt, dass,* we examined 1) whether “CC P, Q” with P entails Q, i.e., whether it would receive high ratings for Q in the MP condition, and 2) whether “CC P, Q” with not-P entails not-Q, i.e., whether it would receive low ratings for Q in the DA condition. If both 1) and 2) hold for *vorausgesetzt, dass*, we take it to be semantically biconditional. Otherwise, it is not biconditional. We also measured the reaction latencies, with no specific predictions.

### Results

**Fig. 5:**
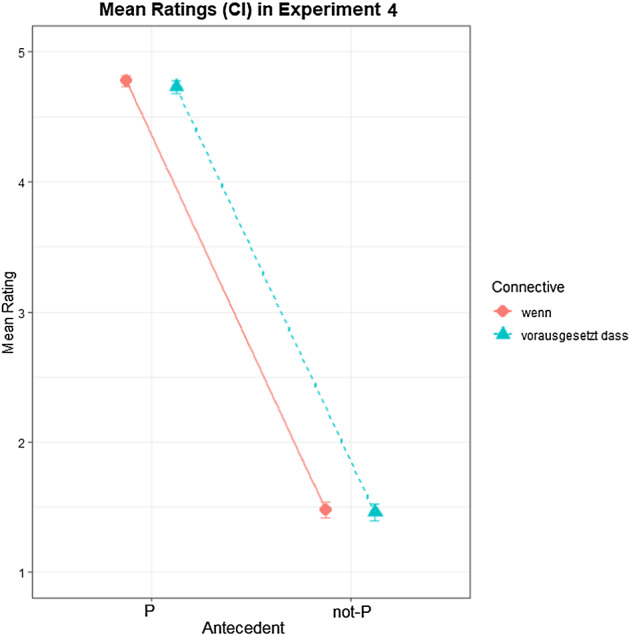
Mean ratings for Q (1 = no, 5 = yes) by condition in Experiment 4. Error bars indicate 90% CI.

Of the 2352 trials, 8 were discarded because ratings were not given within 10 s. Another 44 trials were discarded because their response time deviated more than 3 SDs from their respective subject means. Participant ratings by condition are shown in Fig. 5. A Bayesian ordinal regression model was fitted with CC, Antecedent and their interaction as fixed effects as well as random effects by subject and by item. Both CC and Antecedent were dummy coded, with *wenn* and P as reference levels. Table 7 shows the model output.

**Table 7:** Bayesian regression model output on sentence ratings in Experiment 4

	Estimate	I-95% CI	u-95% CI
*Intercept[1]*	− 9.11	− 10.04	− 8.22
*Intercept[2]*	− 6.61	− 7.54	− 5.76
*Intercept[3]*	− 5.28	− 6.16	− 4.47
*Intercept[4]*	− 2.54	− 3.33	− 1.84
*CC_vd*	− 0.22	− 0.65	0.22
*Antecedent_not-P*	− 10.30	− 11.71	− 9.00
*CC_vd:Antecedent_not-P*	0.04	− 0.55	0.68

Formula = response_ord ~ 1 + CC * Antecedent + (1 + CC * Antecedent|subjectID) + (1 + CC * Antecedent|itemID); Family = cumulative

Hypothesis-specific tests conducted with *brms* (Bürkner, 2017) showed a decisive main effect of Antecedent, with lower ratings for Q in the not-P conditions than in the P conditions (*β* = − 20.56; CI = [− 22.76; − 18.46]; BF_10_ > 3000). Additionally, a small, unreliable main effect of CC was attested, with ratings for *wenn* being non-decisively higher than for *vorausgesetzt, dass* (*β* = − 0.4; CI = [− 0.96; 0.16]; BF_10_ = 7.45). This effect of CC is unreliable in both P (*β* = − 0.22; CI = [− 0.58; 0.14]; BF_10_ = 5.65) and not-P conditions (*β* = − 0.18; CI = [− 0.56; 0.22]; BF_10_ = 3.6), with both Bayes factors below 10. No evidence was found for a significant interaction of CC with Antecedent (*β* = 0.04; CI = [− 0.45; 0.56]; BF_10_ = 0.8). These findings show that ratings for *wenn* and *vorausgesetzt, dass* were not statistically different in both P and not-P conditions, indicating that the two CCs were interpreted similarly.

A Bayesian linear mixed-effects regression model on participants decision latencies was fitted with CC and Antecedent as well as their interaction as fixed effects as well as random effects by subject and by item. Table 8 shows the model output. Besides a decisive main effect of Antecedent (*β* = 356.94; CI = [202.03; 513.55]; BF_10_ = 2141) indicating that response latencies were faster for the P than for the not-P conditions, no main effect of CC (*β* = 16.99; CI = [− 57.62; 92.06]; BF_10_ = 1.82), and no interaction of CC and Antecedent (*β* = 11.46; CI = [− 89.09; 112.73]; BF_10_ = 1.36) were found.

**Table 8:** Bayesian regression model output on rating latencies in ms in Experiment 4

	Estimate	I-95% CI	u-95% CI
*Intercept*	1652.17	1485.64	1820.38
*CC_udBd*	− 3.27	− 98.22	92.01
*Antecedent_not-P*	206.57	99.97	313.40
*CC_udBd:Antecedent_not-P*	22.61	− 139.09	183.49

Formula = responseTime ~ 1 + CC * Antecedent + (1 + CC * Antecedent | subjectID) + (1 + CC * Antecedent|itemID); Family = gaussian

### Discussion

Experiment 4 investigated participants’ ratings for conditional scenarios containing the CC *wenn* (‘if’) or *vorausgesetzt, dass* (‘provided that’) while testing the MP and the DA inferences. The rating patterns for both CCs were found to be statistically identical, which is evidence that *vorausgesetzt, dass* is similar to *wenn* in terms of biconditionality. As with *wenn*, ratings for *vorausgesetzt, dass* were at ceiling for MP, indicating that MP is valid for both CCs. Similarly, ratings for DA were comparable in both CCs, indicating that DA is not a generally valid inference for either of the two CCs. As in Experiment 1, DA ratings (i.e., ratings for Q in DA) for both *wenn* and *vorausgesetzt, dass* were quite low, which reflects frequent pragmatic enrichment of the presented scenarios via conditional perfection.

## Experiment 5

This fourth sentence rating experiment is designed to replicate the critical findings of the sentence completion experiment (Experiment 1) reported in Sect. 3 and to generalize the findings of Experiments 2 and 3 to stimuli that used AC instead of DA inferences. Moreover, this experiment includes an additional complex CC, *im Falle, dass* (‘in the case that’) in order to investigate whether its meaning resembles that of *wenn* or *nur wenn* in terms of biconditionality.

### Method

#### Participants

102 German native speakers (69 female, 3 non-binary, mean age = 21, SD = 3.1) were recruited as paid participants online via Prolific.

#### Materials and design

Forty eight critical items were composed as sets of three sentences each, see (12). Sentence 1 (S1) had the format ‘CC P, Q’, with CC as a factor with four levels being spelled out as *wenn* (‘if’), *im Falle, dass* (‘in the case that’), *unter der Bedingung, dass* (‘on codition that’), or *nur wenn* (‘only if’). S2 presented either the confirmed antecedent or the confirmed consequent (P or Q) in isolation, and S3 asked for the validity of the respective other proposition (Q or P). Hence, the experiment used a 4 × 2 design, with CC (*wenn*/*nur wenn/im Falle, dass/unter der Bedingung, dass*) and Inference (MP vs. AC) as factors. Participants were either asked to rate a set of sentences presenting an inference based on MP (S1: if P, Q. S2: P. S3: Q?), see (12a), or AC (S1: if P, Q. S2: Q. S3: P?), see (12b). On both MP and AC, high ratings indicate high validity of the given inference.

(12)
  - S1: *Wenn heute gutes Wetter ist, geht Kai Eis essen.*
    (If the weather is nice today, Kai will go have ice cream.)
    S2: *Heute ist gutes Wetter.* (The weather is nice today.)
    S3: *Geht Kai Eis essen?* (Will Kai go have ice cream?).
  - S1: *Wenn heute gutes Wetter ist, geht Kai Eis essen.*
    (If the weather is nice today, Kai will go have ice cream.)
    S2: *Kai geht Eis essen.* (Kai will go have ice cream.)
    S3: *Ist heute gutes Wetter ?* (Is the weather nice today?).

The same 48 fillers and 8 practice trials from the previous sentence rating experiment were used here again. Based on a 4 × 2 (CC x Inference) factorial design, 8 counter-balanced experimental lists were designed with equal numbers of trials per condition in each of the lists, so that each item would be presented in only one of the conditions per list. Participants were randomly assigned to one of the lists.

Based on the theoretical assumptions and the experimental findings in Sects. 1–6, we expected that MP inferences are valid for conditionals with *wenn, unter der Bedingung, dass* and possibly *im Falle, dass* but invalid for *nur-wenn*-conditionals, whereas AC inferences are valid for conditionals with *nur wenn* and *unter der Bedingung, dass*, but invalid for *wenn* and *im Falle, dass*. This leads to the prediction of an interaction between CC and Inference: After affirmed consequents (i.e., “CC P, Q” with Q), higher ratings to P are predicted for *nur wenn* and for unt*er der Bedingung, dass* than for the other two CCs; after positive antecedents (i.e., “CC P, Q” with P), lower ratings to Q are predicted for *nur wenn* than for the other three CCs. Furthermore, we also measured reaction latencies, with no specific predictions.

#### Procedure

Each trial began with a fixation cross in the middle of the sentence to appear and each of the sentences replaced the preceding one in the same position. Participants were instructed to read each of the three sentences in each trial carefully at their own pace and press the space bar whenever they were ready to see the next sentence. They were further instructed to quickly answer the final question based on their intuition with one of the following five options by clicking on the chosen answer in the following set of answers that was presented below the question in S3: *Nein.* (no), *Eher nein.* (rather no), *Kann ich nicht sagen* (I cannot tell), *Eher ja.* (rather yes), and *Ja.* (yes). The whole testing session took about 30 min.

### Results

**Fig. 6:**
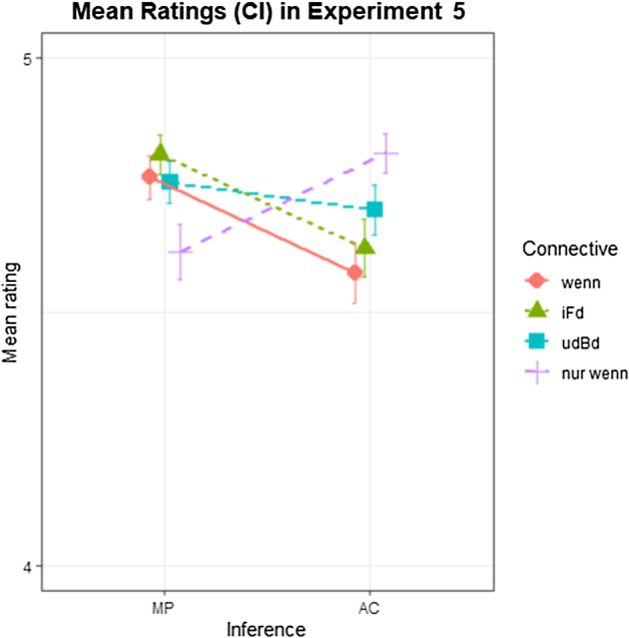
Mean ratings (1 = no, 5 = yes) by condition for Experiment 5. Error bars indicate 90% CI.

Data from two of the 102 participants were excluded because they did not attend to the task, which was obvious by grand mean reaction times below 1000 ms and random response patterns, especially in filler trials. Of the 4800 trials, 43 were discarded because ratings were not given within 10 s. Further, 95 trials were discarded since their decision latencies were outliers of more than 3 SDs by subject. Hence, the final data set contained 4662 trials. Grand mean ratings were at 4.7 (CI: [4.69; 4.72]). Mean ratings by condition are shown in Fig. 6. A Bayesian ordinal regression model was fitted with CC, Inference and their interaction as fixed effects as well as random effects by subject and by item. CC was dummy coded, with *wenn* as reference level; Inference was deviation coded (MP: -0.5; AC: 0.5). Table 9 shows the model output.

**Table 9:** Bayesian regression model output on sentence ratings in Experiment 5

	Estimate	l-95% CI	u-95% CI
*Intercept[1]*	− 7.62	− 8.39	− 6.87
*Intercept[2]*	− 7.25	− 7.97	− 6.54
*Intercept[3]*	− 4.73	− 5.33	− 4.15
*Intercept[4]*	− 2.03	− 2.59	− 1.47
*CC_iFd*	0.45	0.16	0.78
*CC_udBd*	0.73	0.29	1.26
*CC_nurwenn*	0.40	0.07	0.74
*Inference*	− 0.90	− 1.55	− 0.24
*CC_iFd:Inference*	0.03	− 0.56	0.63
*CC_udBd:Inference*	1.10	0.48	1.73
*CC_nurwenn:Inference*	2.47	1.57	3.37

Population-Level Effects. Formula = response_ord ~ 1 + CC * Inference + (1 + CC * Inference|subjectID) + (1 + CC * Inference|itemID); Family = cumulative

Hypothesis-specific tests in *brms* showed a decisive effect of Inference in *wenn* (*β* = -0.90; CI = [− 1.55; − 0.24]; BF_10_ = 213), with ratings for AC being lower than for MP. A comparison of the effect of Inference in *wenn* with the effect of Inference in *im Falle, dass* shows that the two CCs behave equivalently (*β* = 0.03; CI = [− 0.45; 0.54]; BF_10_ = 1.2). The effect of Inference in *unter der Bedingung, dass* is, however, decisively smaller (*β* = 1.1; CI = [0.58; 1.62]; BF_10_ = 1499), as *unter der Bedingung, dass* does not show a reliable effect of Inference (*β* = 0.2; CI = [− 0.39; 0.82]; BF_10_ = 2.3). *Nur wenn* shows a decisive effect of Inference (*β* = 1.77; CI = [1.07; 2.51]; BF_10_ = > 3000), which is decisively different from *wenn*, going in the opposite direction (*β* = 1.57; CI = [0.91; 2.22]; BF_10_ > 3000), with ratings for AC being higher than for MP.

**Fig. 7:**
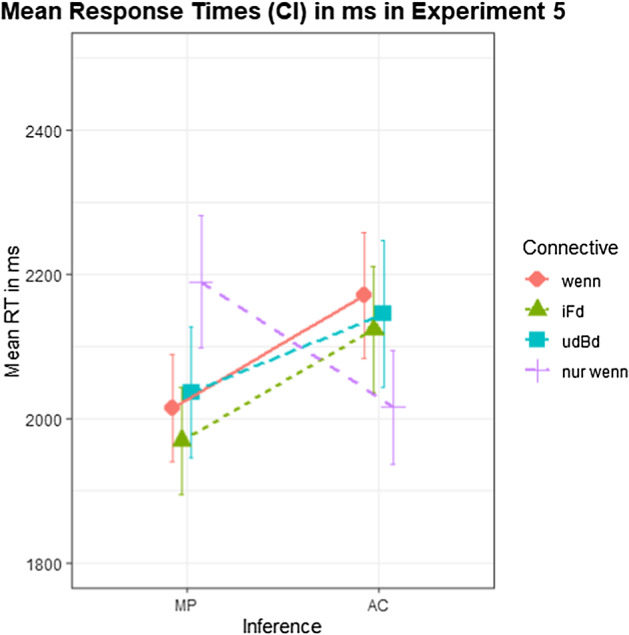
Mean response latencies by condition for Experiment 5. Error bars indicate 90% CI.

Grand mean decision latencies were 2082 ms (CI: [2051 ms, 2112 ms]). Mean latencies by condition are shown in Fig. 7. A Bayesian linear mixed-effects regression model was fitted with CC, Inference and their interaction as fixed effects as well as as random effects by subject and by item. CC was dummy coded, with *wenn* as reference level; Inference was deviation coded (MP: -0.5; AC: 0.5). Table 10 shows the model output.

**Table 10:** Bayesian regression model output on decision latencies in Experiment 5

	Estimate	l-95% CI	u-95% CI
*Intercept*	2101.00	1980.25	2215.96
*CC_iFd*	− 57.03	− 132.46	17.87
*CC_udBd*	− 3.25	− 90.02	81.54
*CC_nurwenn*	12.32	− 66.64	92.09
*Inference*	166.47	54.22	280.17
*CC_iFd:Inference*	− 3.39	− 153.97	147.44
*CC_udBd:Inference*	− 43.68	− 213.13	122.23
*CC_nurwenn:Inference*	− 346.28	− 544.27	− 149.69

Population-Level Effects. Formula = responseTime ~ 1 + CC * Inference + (1 + CC * Inference|subjectID) + (1 + CC * Inference|itemID); Family = gaussian

Hypothesis-specific tests in *brms* showed a decisive effect of Inference in *wenn* (*β* = 166.47; CI = [71.97; 261.42]; BF_10_ = 516), with decisions in AC being slower than in MP. This effect is equally strong in both *im Falle, dass* (*β* = − 3.39; CI = [− 130.46; 124]; BF_10_ = 1.05) and *unter der Bedingung, dass* (*β* = − 43.68; CI = [− 184.73; 95.88]; BF_10_ = 2.3). In *nur wenn*, the effect of Inference goes in the opposite direction (*β* = − 179.81; CI = [− 316.69; − 47.78]; BF_10_ = 72.53), with decision latencies in AC being shorter than in MP.

### Discussion

With this experiment, we tested the effects of different CCs on validity ratings in MP and AC inferences. While MP is valid for both simple conditionals (P → Q) and biconditionals (P ↔ Q), AC is only valid for biconditionals. This experiment tested four German CCs on their biconditionality. While all conditionals got relatively high ratings in both MP as well as AC conditions, the simplest connective *wenn* showed a strong effect of Inference, receiving lower ratings in the AC condition than in the MP condition. Ratings in *im Falle, dass* were parallel to ratings in *wenn*, indicating that these two connectives are very similar in terms of semantic biconditionality; this does not mean that they are similar in meaning in general though, since, for example, *wenn* has a temporal meaning that *im Falle, dass* lacks. Ratings in *unter der Bedingung, dass*, however, showed a smaller effect of Inference than *wenn* or *im Falle, dass*, if any, indicating that *unter der Bedingung, dass* received biconditional interpretations more often than the latter two connectives. Consequently, *unter der Bedingung, dass* is, in comparison, the best candidate for a natural language biconditional connective.

AC ratings in *nur wenn* were higher than in the other CCs. Even more interestingly, MP ratings in *nur wenn* were lower than MP ratings in *wenn*, and also lower than AC ratings in *nur wenn*. These results are in line with the findings of Experiments 1 and 2. They suggest that *nur wenn* is not interpreted as a biconditional connective, since MP ratings (i.e., the consequent becoming true given the antecedent) are reduced in *nur wenn* compared to *wenn*.

A central open question concerns the generally high mean AC ratings, indicating that in all of the tested CCs, AC is valid in many of the tested scenarios, while, logically speaking, they called for ‘*I cannot tell*’ judgements. One reason might lie in the phrasing ‘*I cannot tell*’, which might feel like a failure to participants and might therefore be avoided, especially since the most probable of cases in the simplistic scenarios that were presented in the three given sentences is that the antecedent is true and is the reason for the consequent to also be true. Indeed, in a follow-up replication experiment focusing on *wenn* and using an alternative phrasing for the neutral response option (“*This is uncertain*” instead of “*I cannot tell*”), we found that mean AC ratings were significantly reduced, while ratings for MP were unaffected. Details of the methods and results of this replication experiment can be found in the Appendix.

## General Discussion and Conclusion

In this paper, we conducted four studies testing the biconditionality of German conditional connectives (CCs). In an initial sentence production task, we collected balanced data for the three CCs *wenn, nur wenn* and *wenn und nur wenn*. The qualitative analysis of the data indicates that *wenn und nur wenn* is semantically biconditional, but that *wenn* and *nur wenn* are not. As *wenn und nur wenn* is rarely used in German, in Experiment 1, using a sentence completion task, we zoomed in on the meaning of *wenn* vs. *nur wenn*. The results provide first experimental evidence that neither *wenn* nor *nur wenn* is biconditional: for *wenn*, not all scenarios related to DA were completed with DA-endorsing responses; for *nur wenn*, not all scenarios related to MP were completed with MP-endorsing responses. In Experiment 2, using a sentence rating task where we compared ratings on MP and DA arguments in simple scenarios, the pattern found in Experiment 1 was confirmed. MP was endorsed in *wenn* but not always in *nur wenn*, while DA was endorsed in *nur wenn* but not always in *wenn*. Using the same method as in Experiment 2, we tested *unter der Bedingung, dass* vs. *wenn* and found that they were similar with regard to the validity of the MP inference but the former received higher ratings with regard to the validity of the DA inference. Experiment 3 tested *vorausgesetzt, dass* vs. *wenn* and did not find any difference between them in the MP or DA inference. In Experiment 5, another sentence rating task using AC inferences and adding two more complex CCs, we replicated the results of Experiment 2 for *nur wenn* vs. *wenn.* Furthermore, we found the third tested CC *im Falle, dass* (‘in the case that’) to be interpreted similarly to *wenn.* The fourth tested CC *unter der Bedingung, dass* (‘on condition that’), however, was found to be the most promising candidate for a biconditional CC, as it demonstrates symmetry in terms of MP and AC inferences in comparison to the other three CCs, which showed asymmetry in this aspect. The results are summarized in Table 11—Note though, that whereas our data show tentative evidence for the biconditionality for *unter der Bedingung, dass*, further research is still needed for confirming this conclusion.

**Table 11:** Summary of results of experiments 1–4

Experiment	Measures	CC	MP	DA	AC
1	Sentence completion	*wenn*	Valid	Invalid	
		*nur wenn*	Invalid	Valid	
2	Sentence rating	*wenn*	Valid	Invalid	
		*nur wenn*	Invalid	Valid	
3	Sentence rating	*wenn*	Valid	Invalid	
		*unter der Bedingung, dass*	Valid	Valid	
4	Sentence rating	*wenn*	Valid	Invalid	
		*vorausgesetzt, dass*	Valid	Invalid	
5	Sentence rating	*wenn*	Valid		Invalid
		*nur wenn*	Invalid		Valid
		*unter der Bedingung, dass*	Valid		Valid
		*im Falle, dass*	Valid		Invalid

The results show that neither *wenn* nor *nur wenn* are semantically biconditional, but for different reasons. The findings about *wenn* can be accounted for based on material implication and conditional perfection: The former explains the validity of the MP and the invalidity of the DA or AC inference. The latter explains why the DA or AC inference was judged as valid in some (though not all) of the tested scenarios, as the invited inference is pragmatic in nature and thus context-dependent (van Canegem-Ardijns & Van Belle, 2008). One potential factor for the context-dependency that has been discussed in the literature concerns questions under discussion (QuDs). Von Fintel (2011) and Arregui and Biezma (2016) argue that conditionals are related to two different kinds of QuDs, namely *“Under which conditions Q?” or “What follows from P?”* and that conditional perfection is triggered when a conditional sentence functions as an exhaustive answer to the former QuD (see Cariani & Rips, 2018 for related psycholinguistic studies). In addition to pragmatic context, whose effect remains to be further investigated, our study with the *wenn/nur wenn* contrast as well as their comparisons with the other tested CCs (i.e., *vorausgesetzt, dass* / *im Falle, dass* / *unter der Bedingung, dass*) in Experiments 3, 4 and 5 shows that different CCs can differ in this aspect as well. For example, the inference of not-P→not-Q, which is invited for “Wenn P, Q”, is a semantic meaning component for “Nur wenn P, Q”. On the other hand, “Nur wenn P, Q” does not semantically encode the inference of P→Q, which *wenn*-conditionals do. This raises the question whether the latter meaning component can be invited for *nur-wenn*-sentences as well, what contextual constraints exist, and if yes, how the context-dependency of this invited inference differs from conditional perfection in the classical sense of simple conditionals. An additional open question is why conditional perfection arises in most of the tested cases. While we are not able to provide any definitive answer, a possible reason is that in the tested scenarios the antecedent was not only a very plausible condition but also the only mentioned one in the context, that is, there are no other contextual alternatives. This might have invited the comprehenders to take the given antecedent as both a necessary and sufficient condition for the consequent.

The findings about *nur wenn,* regarding the DA or AC inference, are in line with the formal perspective that entails the negative proposition *if not-P, not-Q*. In contrast, the MP inference did not bear out as predicted by a compositional analysis resting on the treatment of bare conditionals as involving universal quantification. Instead, the result provides convergent supporting evidence for Herburger’s (2015, 2019) analysis assuming that *only-if-*sentences such as “*Only if you work hard do you succeed.*” encode the semantics that some (not all, as in the case of bare *if*-conditionals) instances of hard work will be rewarded by success. The lack of biconditionality in *nur wenn* or *only if* is compatible with the minimal pair in (13) from Horn (1996), showing that the continuation of not-Q is possible given “Only if P, Q.” and P, whereas it is odd for biconditionals.

(13)
  - * I’ll go only if you go, and maybe not even then.*
  - * #I’ll go if and only if you go, and maybe not even then.*

In addition to theoretical implications about the lexical meaning of the tested CCs, this study also has methodological implications. For example, in German, *nur* does not need to be adjacent to *wenn* but can occur in the consequent (14a). Furthermore, Andreas Blümel (p.c.) pointed out to us that (14b) with stress on *dann* (‘then’) has a biconditional reading. In logic, *iff* is often translated as “*genau dann, wenn*” (‘exactly then, if’), see (14c). While we did not study these related expressions, the experimental paradigms we used in the paper can in principle be applied to these cases to test their semantics, as well as to the other conditionals in different languages.

(14)
  - *Ich werde nur schlafen gehen, wenn du gehst.*
    (I will only go to sleep if you leave.)
  - *Ich werde DANN schlafen gehen, wenn du gehst.*
    (If and only if you leave, I will go to sleep.)
  - *Ich werde genau dann schlafen gehen, wenn du gehst.*
    (If and only if you leave, I will go to sleep.)

Before we end, we would like to briefly address the scope and limitation of the current study. First, in this paper, we focused on a small set of CCs in German in terms of semantic biconditionality. The results we obtained are certainly contingent on, for example, the specific experimental design, the specific CCs and the specific contexts used in the scenarios. The variation among the long list of CCs as in (1) in this aspect or others awaits further research. Secondly, conditionals are notorious for their context-dependent interpretations. We were not able to deal with the question how the semantic and pragmatic properties of CCs interact with other expressions in the sentence or the broad discourse context. However, the finding of differences between different CCs open up novel perspectives on the interpretation and processing of conditionals in general. CCs, while being an essential component in many conditional sentences, are understudied in psycholinguistic research – our study provides first steps to understand their effects on the interpretation and processing of conditionals.

## Figures and Tables

**Fig. 8:**
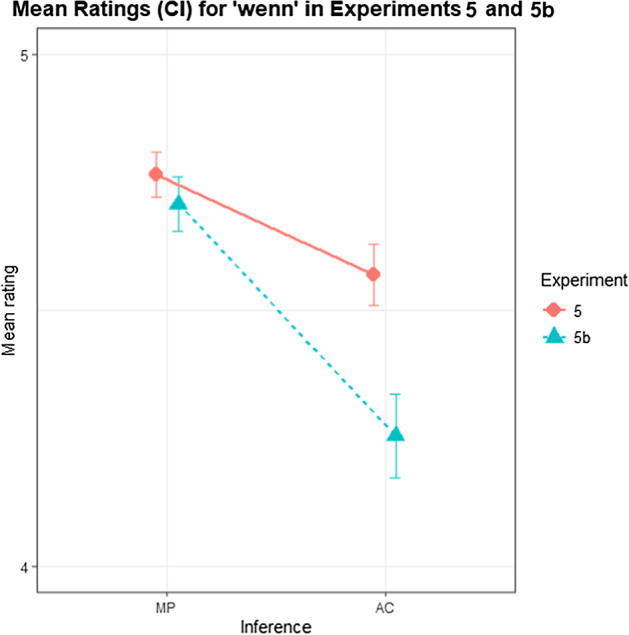
Ratings (1 = no, 5 = yes) in sentence rating Experiments 5 and 5b by response category. Error bars show 90% CI.

**Fig. 9:**
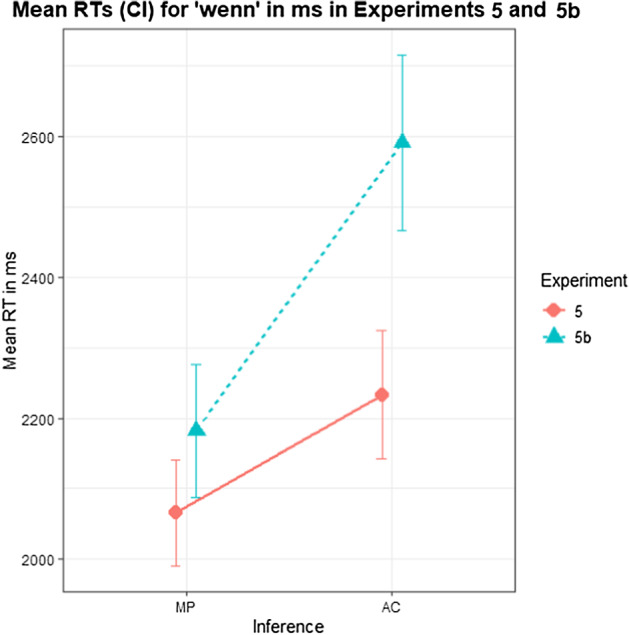
Decision latencies in sentence rating Experiments 5 and 5b by response category. Error bars show 90% CI.

## Data Availability

All data and code associated with the experiment reported in this paper are available through this link to an (anonymised) data repository: https://osf.io/unq3s/

## Experiment 5b

### Method

#### Participants

Twenty three German native speakers (17 female, 1 non-binary, mean age = 27, SD = 7.5) that did not take part in the previous experiments were recruited via Prolific as paid participants online.

## Materials and Design

The same 48 critical items used in Experiment 5 were used and all of them were tested with the CC *wenn* in S1. S2 and S3 again presented either Modus Ponens (MP) or Affirmation of the Consequent (AC) arguments. The same set of 48 filler items and 8 practice trials used in Experiment 5 was used here. 2 counter-balanced experimental lists were designed with equal numbers of trials per condition in each of the lists. Participants were randomly assigned to one of the lists.

## Procedure

The same procedure as in Experiment 5 was used, with the crucial difference that the wording of the neutral response option 3 was changed to ‘*Das ist nicht sicher*’ (That's not sure.). The whole testing session took about 30 min.

## Results

Data from one of the 23 participants were excluded due to grand mean reaction times below 1000 ms and random response patterns. In total, 81 critical trials (7.7%) were discarded because reading times in Sentences 1 or 2 were below 300 ms, reading times in S2 were above 10 s, or because decision latencies were longer than 10 s or shorter than 1 s (including reading the question). Further, 20 trials were discarded since their decision latencies were outliers of more than 3 SDs by subject. Hence, the final data set contained 955 trials.

A Bayesian ordinal mixed-effects model was fitted with Inference as the single fixed effect and as random effect by subject and by item (see Table

**Table 12:** Model output of sentence rating in Experiment 5b

	Estimate	l-95% CI	u-95% CI
*Intercept[1]*	− 6.79	− 7.94	− 5.72
*Intercept[2]*	− 6.21	− 7.22	− 5.26
*Intercept[3]*	− 2.78	− 3.53	− 2.10
*Intercept[4]*	− 1.04	− 1.73	− 0.38
*Inference*	− 1.71	− 3.04	− 0.48

Formula = response_ord ~ 1 + Inference + (1 + Inference | subjectID) + (1 + Inference | itemID); Family = cumulative

12). Inference was deviation coded (MP:− 0.5; AC: 0.5). A decisive effect of Inference was attested (*β* = 170.16; CI = [77.27; 261.66]; BF_10_ = 936), with ratings being lower in AC than in MP. Figure 8 shows a comparison of the effect of Inference between Experiments 5 and 5b. While MP ratings were comparable to Experiment 5, AC ratings were considerably lower, with complete acceptance at 55% and the neutral responses category 3 being chosen 12% of the time.

A Bayesian linear mixed model fitting reaction times was run with Inference as a fixed effect and as a random effect by subject and by item (see Table

**Table 13:** Model output on decision latencies in Experiment 5b

	Estimate	l-95% CI	u-95% CI
*Intercept*	2355.27	2095.87	2609.02
*Inference*	400.58	161.45	640.03

Formula = responseTime ~ 1 + Inference + (1 + Inference | subjectID) + (1 + Inference | itemID); Family = gaussian

13). Inference was found to have a decisive effect on decision latencies (*β* = 400.58; CI = [200.79; 600.28]; BF_10_ = 516), with decisions being slower in AC than in MP. A comparison of reaction times in *wenn* between Experiments is shown in Fig. 9.

## Discussion

The effect of Inference attested in Experiment 5 was replicated. While the effect was rather small in the previous experiment, both in ratings and reaction times, the effects are much more pronounced in the replication, where participants seriously considered the neutral response option 3, as was reflected in much higher ratings of this option and significantly slower reaction times in AC as compared to the previous experiment. These differences show that acceptability judgements are highly dependent on methodological details such as the phrasing of the response options.
